# Identification of Bisindolylmaleimide IX as a potential agent to treat drug-resistant BCR-ABL positive leukemia

**DOI:** 10.18632/oncotarget.11566

**Published:** 2016-08-24

**Authors:** Xin Zhang, Deyong Jia, Junping Ao, Huijuan Liu, Yi Zang, Mohammad Azam, Samy L. Habib, Jia Li, Xinsen Ruan, Hao Jia, Xueying Wang, Baojie Li

**Affiliations:** ^1^ Bio-X Institutes, Key Laboratory for the Genetics of Developmental and Neuropsychiatric Disorders, Ministry of Education, Shanghai Jiao Tong University, Shanghai, China; ^2^ State Key Laboratory of Oncogenes and Related Genes, Shanghai Cancer Institute, Renji Hospital, Shanghai Jiao Tong University, School of Medicine, Shanghai, China; ^3^ National Center for Drug Screening, Shanghai Institute of Materia Medica, Shanghai Institutes for Biological Sciences, Chinese Academy of Sciences, Shanghai, China; ^4^ Divisions of Pathology, Hematology and Cancer Biology, Cancer and Blood Disease Institute, Cincinnati Children's Hospital and Medical Center, Cincinnati, OH, USA; ^5^ South Texas Veterans Health Care System and Department of Cellular and Structural Biology, University of Texas Health Science Center at San Antonio, San Antonio, TX, USA; ^6^ Department of Biochemistry, National University of Singapore, Singapore; ^7^ Translational Medical Center for Stem Cell Therapy, Shanghai East Hospital, Tongji University School of Medicine, Shanghai, China

**Keywords:** Bisindolylmaleimide IX, BCR-ABL, chronic myeloid leukemia (CML)

## Abstract

Chronic myeloid leukemia (CML) treatment with BCR-ABL inhibitors is often hampered by development of drug resistance. In a screen for novel chemotherapeutic drug candidates with genotoxic activity, we identified a bisindolylmaleimide derivative, IX, as a small molecule compound with therapeutic potential against CML including drug-resistant CML. We show that Bisindolylmaleimide IX inhibits DNA topoisomerase, generates DNA breaks, activates the Atm-p53 and Atm-Chk2 pathways, and induces cell cycle arrest and cell death. Interestingly, Bisindolylmaleimide IX is highly effective in targeting cells positive for BCR-ABL. BCR-ABL positive cells display enhanced DNA damage and increased cell cycle arrest in response to Bisindolylmaleimide IX due to decreased expression of topoisomerases. Cells positive for BCR-ABL or drug-resistant T315I BCR-ABL also display increased cytotoxicity since Bisindolylmaleimide IX inhibits B-Raf and the downstream oncogene addiction pathway. Mouse cancer model experiments showed that Bisindolylmaleimide IX, at doses that show little side effect, was effective in treating leukemia-like disorders induced by BCR-ABL or T315I BCR-ABL, and prolonged the lifespan of these model mice. Thus, Bisindolylmaleimide IX presents a novel drug candidate to treat drug-resistant CML via activating BCR-ABL-dependent genotoxic stress response and inhibiting the oncogene addiction pathway activated by BCR-ABL.

## INTRODUCTION

Cancer is a leading cause of mortality worldwide and can be treated with radiotherapy and/or chemotherapy [1]. Great efforts have been taken to develop new chemotherapy agents due to resistance, insufficient efficacy and/or side effects of the available drugs [2]. For example, CML is mainly caused by BCR-ABL, a constitutively active tyrosine kinase generated by chromosome translocation [3, 4]. CML is also an oncogene addiction model, which provides the rationale to target BCR-ABL or its downstream oncogene addiction pathways [5, 6]. Imatinib, an ABL kinase inhibitor, is effective for initial treatment of CML, yet a large percentile of CML patients gradually develop resistance [7], due to mutations in BCR-ABL, e.g., T315I, which disrupt imatinib-BCR-ABL interaction [8]. The occurrence of these mutations is driven by BCR-ABL itself, as BCR-ABL promotes DNA damage via reactive oxygen species (ROS)-dependent and -independent mechanisms [9–11], and affects multiple DNA repair processes [12, 13]. Most of the imatinib-resistant BCR-ABL mutants are sensitive to the next-generation drugs nilotinib and dasatinib [14], with the exception of T315I BCR-ABL mutant. As a result, new drugs that can overcome the resistance are needed to combat CML [15–17].

Many of the chemotherapeutic agents are genotoxic and can cause genome instability in tumor cells as well as normal cells [2]. Cells respond to DNA damage by activating the PI-3 kinase-like kinases (PIKKs), including Atm and Atr, at the DNA break sites, where they phosphorylate substrates including H2AX, Chk1, Chk2, and p53. Activation of Chk1 and Chk2 via phosphorylation induces G2/M arrest and S phase delay, while activation of p53 causes G1 and G2/M arrest and/or programmed cell death [18, 19]. Thus DNA damage response (DDR) leads to cease of cell propagation in p53-dependent and -independent manners [20], which is also the main mechanism by which genotoxic agents restrain cancer growth [2], even in tumors that express mutant p53 molecules with loss-of-function or dominant negative effects [21–23].

Here we report the identification of Bisindolylmaleimide IX as a genotoxic agent with potential to treat CML. Bisindolylmaleimide IX is a member of the bisindolylmaleimide compounds that were initially synthesized as PKC inhibitors and later were shown to have inhibitory effects on a spectrum of kinases [24, 25]. One derivative, Enzastaurin (LY317615), has been tested alone or in combination with other chemotherapeutic drugs in multiple clinical trials to treat various types of cancer [26, 27]. This study reveals that Bisindolylmaleimide IX is a DNA topoisomerase inhibitor and an inhibitor of B-Raf. While it generates DNA damage and activates the DNA damage response in all cells tested, it induces enhanced DNA damage in BCR-ABL positive cells, likely due to reduced levels of topoisomerase II isoforms, and causes increased cell cycle arrest in these cells. Furthermore, Bisindolylmaleimide IX also shows increased cytotoxicity to BCR-ABL positive cells by inhibiting the oncogene addiction Raf-Erk pathway [6, 28]. Importantly, Bisindolylmaleimide IX is effective in treating CML-like leukemia caused by BCR-ABL or T315I mutant BCR-ABL. These findings suggest that Bisindolylmaleimide IX has the potential to treat multi-drug resistant CML. The effective doses of Bisindolylmaleimide IX are similar to those of topoisomerase inhibitors such as doxorubicin and show little side effect in vivo. The study thus identified a prototype for new anti-CML drug design.

## RESULTS

### Identification of Bisindolylmaleimide IX as a genotoxic drug

**Figure 1:**
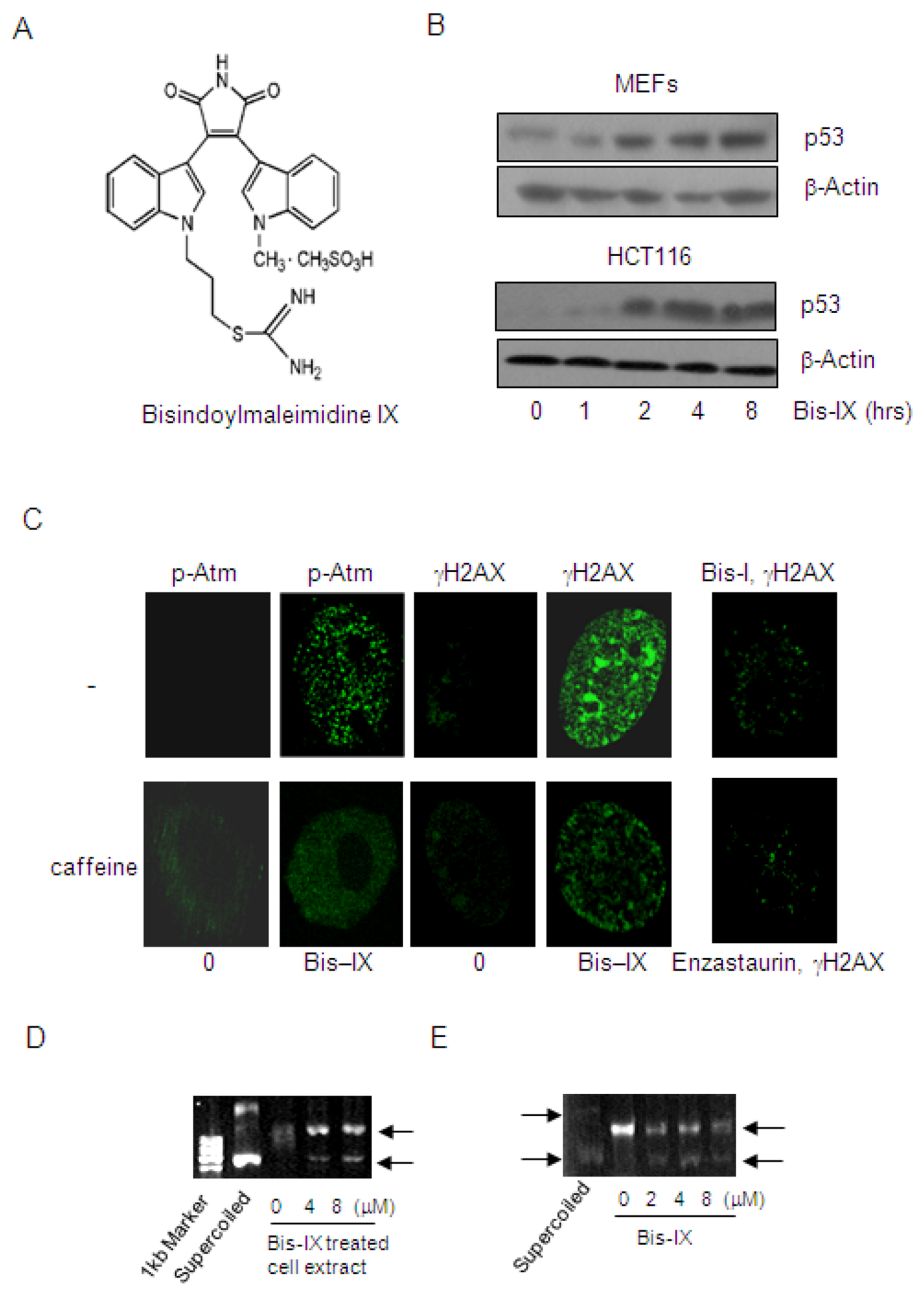
Identification of Bisindolylmaleimide IX as a genotoxic agent and a topoisomerase inhibitor **A.** The structures of Bisindolylmaleimide IX. **B.** Bisindolylmaleimide IX induced p53 expression in MEFs and HCT116 cells. Upper panel: primary MEFs were treated with 2.5 μM Bisindolylmaleimide IX for different periods of time and the cells were collected. The levels of p53 were determined by western blot. Bottom panel: HCT116 cells were treated with 2.5 μM Bisindolylmaleimide IX for different periods of time and the cells were collected. The levels of p53 were determined by western blot. **C.** Bisindolylmaleimide IX induced formation of DNA damage foci for γH2AX and p-Atm in MEFs. Primary MEFs were pretreated with caffeine or solvent for 2 hrs and then with 2.5 μM of Bisindolylmaleimide IX, Bisindolylmaleimide I, or Enzastaurin for 4 more hrs. γH2AX and p-Atm were detected with immunofluorescent staining using specific antibodies. **D.** Bisindolylmaleimide IX inhibited the topoisomerase activity assayed with pBluescript. DNA samples of pBluescript were incubated with cell lysates of MEFs, which were treated with 0, 4 or 8 μM Bisindolylmaleimide IX before being harvested. DNA samples were analyzed on agarose gels. **E.** Bisindolylmaleimide IX directly inhibited the topoisomerase activity in vitro assays. DNA samples of pBluescript were incubated with cell lysates of BaF3 in the presence of 0, 2, 4 or 8 μM Bisindolylmaleimide IX. DNA samples were analyzed on agarose gels.

To search for novel anti-cancer agents with genotoxic activities, we screened a panel of kinase inhibitors and a panel of phosphatase inhibitors using p53 protein as an initial indicator of DNA damage. Primary mouse embryonic fibroblasts (MEFs) were chosen for the screen since many cancer cell lines have altered DNA damage response, in particular the loss-of-function mutations of p53 [29, 30]. Among the 113 small molecule compounds, two were found to up-regulate p53 at the protein levels. One is 5-Iodotubercidin (Itu) and the other is Bisindolylmaleimide IX (Figure 1A and 1B). We have reported that 5-Iodotubercidin, as a nucleoside analog that can be incorporated into DNA and cause DNA damage, is effective in treating MEF or HCT116-induced tumors in mouse models [31]. Bisindolylmaleimide IX (3-[1-[3-(Amidinothio)propyl]-3-indolyl]-4-(1-methyl-3-indolyl)-1H-pyrrole-2,5- dione methanesulfonate, Ro-31-8220) is a member of the bisindolylmaleimide derivatives and a cell permeable inhibitor for PKC isoforms including PKC-α, PKC-βI, PKC-βII, PKC-γ, and PKC-ε (Supplementary Figure S1). One previous study reported that Bisindolylmaleimide IX could up-regulate p53 via PKC [32]. However, we found that all the other PKC inhibitors in the kinase inhibitor panel including GF109203X (Bisindolylmaleimide I), H-7, H-9, staurosporine, Hypericin, Rottlerin, Sphingosine, Palmitoyl-DL-carnitine Cl, HBDDE (2,2′,3,3′,4,4′-Hexahydroxy-1,1-biphenyl-6,6′-dimethanol Dimethyl Ether) failed to up-regulate p53 in MEFs, suggesting that PKC kinases may not be the main reason behind p53 induction elicited by Bisindolylmaleimide IX. In addition to MEFs, Bisindolylmaleimide IX was able to induce p53 expression in a human colon cancer line HCT116 in a time-dependent manner (Figure 1B).

### Bisindolylmaleimide IX inhibits DNA topoisomerases

Genotoxic stress is a major activator of p53. To confirm that Bisindolylmaleimide IX is a genotoxic agent, we first looked at γH2AX, an indication of DNA breaks [22], and found that Bisindolylmaleimide IX induced formation of numerous foci positive for γH2AX in MEFs, whereas two other bisindolylmaleimide derivatives Enzastaurin and Bisindolylmaleimide I only induced minimal numbers of foci (Figure 1C). γH2AX is a product of Atm/Atr [33]. Inhibition of Atm and Atr with caffeine could diminish γH2AX foci formation (Figure 1C). These results, taken together, suggest that Bisindolylmaleimide IX is a genotoxic agent.

A previous study reported that Bisindolylmaleimide I, when conjugated to lexitropsins but not by itself, showed inhibitory activity against DNA topoisomerase I [34]. We then tested whether Bisindolylmaleimide IX had any effect on DNA topoisomerases using supercoiled pBluescript plasmid DNA as a template and found that while the untreated cell extract showed DNA relaxation activity, cell extract from Bisindolylmaleimide IX-treated MEFs partially lost this activity (Figure 1D). This was confirmed using a topoisomerase relaxation assay kit (Supplementary Figure S2A). However, Bisindolylmaleimide I, XI, Go6976, and Enzastaurin did not show such an effect (Supplementary Figure S2B). Moreover, addition of Bisindolylmaleimide IX to the in vitro DNA relaxation reaction could also inhibit the topoisomerase activity (Figure 1E), suggesting that Bisindolylmaleimide IX may directly, rather than via its metabolites, inhibit DNA topoisomerases and thus cause DNA damage. Topoisomerases are important targets for cancer chemotherapy [35]. Some of the inhibitors including doxorubicin induce DNA damage in cell cultures at doses that are similar to the effective doses of Bisindolylmaleimide IX [35, 36]. Moreover, the expression levels of topoisomerases in tumors also determine chemo-sensitivity to these drugs [36].

### Bisindolylmaleimide IX induces modest cell death and activates cell cycle checkpoints

**Figure 2:**
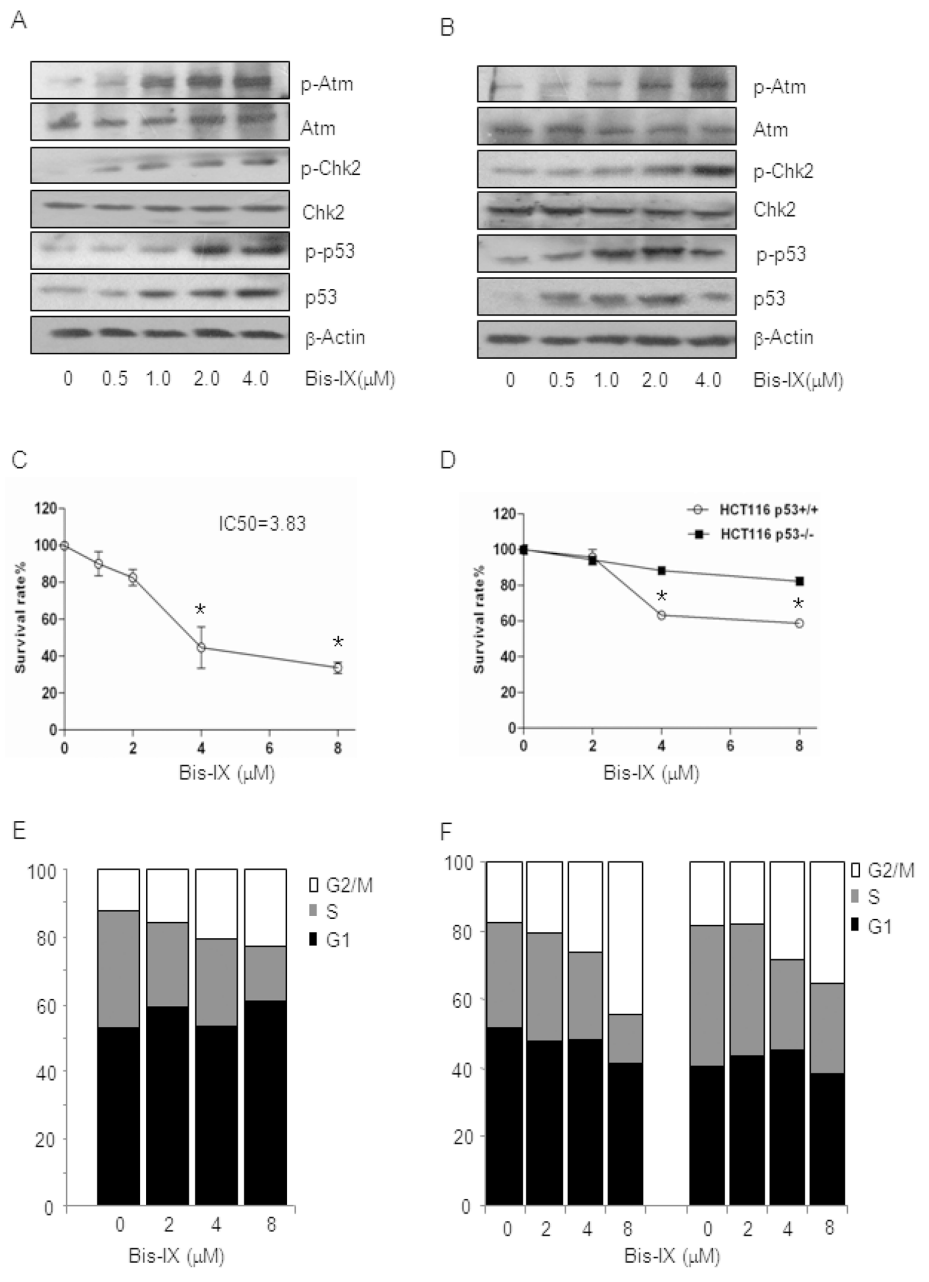
Bisindolylmaleimide IX activated the DNA damage response pathway and induced cell death and cell cycle arrest **A.** Bisindolylmaleimide IX activated the Atm-Chk2 pathway in MEFs in a dose-dependent manner. Primary MEFs were treated with different doses of Bisindolylmaleimide IX for 4 hrs and the cells were collected. The levels of p53, p-p53 (S15), Atm, p-Atm (S1981), Chk2, p-Chk2 (T68) and β-actin were determined by western blot. **B.** Bisindolylmaleimide IX activated the Atm-Chk2 pathway in HCT116 cells in a dose-dependent manner. HCT116 cells were treated with different doses of Bisindolylmaleimide IX for 4 hrs and the cells were collected. The levels of p53, p-p53 (S15), Atm, p-Atm (S1981), Chk2, p-Chk2 (T68), and β-actin were determined by western blot. **C.** Bisindolylmaleimide IX induced cell death in a dose-dependent manner in MEFs. Primary MEFs were treated with different doses of Bisindolylmaleimide IX for 24 hrs and the cell survival rates were determined by Wst-1 assay. N=3. *p<0.05 when the values of treated cells were compared to that of untreated cells. **D.** Bisindolylmaleimide IX induced cell death in HCT116 cells in a p53-dependent manner. p53+/+ or p53−/− HCT116 cells were treated with different doses of Bisindolylmaleimide IX for 24 hrs and the cell survival rates were determined by Wst-1 assay. N=3. *p<0.05 when the values of p53−/− cells were compared to those of p53+/+ cells. **E.** Bisindolylmaleimide IX induced cell cycle arrest in a dose-dependent manner in MEFs. Primary MEFs were treated with different doses of Bisindolylmaleimide IX for 24 hrs and the cell cycle profiles were analyzed with FACS. The values are average of three repeated experiments. **F.** Bisindolylmaleimide IX induced cell cycle arrest in HCT116 cells in a p53-dependent manner. p53+/+ or p53−/− HCT116 cells were treated with different doses of Bisindolylmaleimide IX for 24 hrs and the cell cycle profiles were analyzed with FACS. The values are average of three repeated experiments.

Genotoxic stress usually induces apoptosis via p53 and cell cycle arrest via p53 and Chk1/2, which are downstream of Atm/Atr. We found that Bisindolylmaleimide IX, in a dose dependent manner, activated Atm and Chk2 in MEFs and HCT116 cells, justified by specific phosphorylation of these proteins and formation of foci positive for p-Atm (Figure 1C, 2A, and 2B). As low as 1.0 μM Bisindolylmaleimide IX was able to activate the DNA damage response. We then evaluated the cytotoxic effect of Bisindolylmaleimide IX in MEFs and HCT116 cells. Different doses of Bisindolylmaleimide IX were applied to the cell cultures and cell survival rates and IC_50_ were determined with Wst-1 assay. We found that Bisindolylmaleimide IX seemed to precipitate in cell cultures at concentrations greater than 10 μM. For cells that could not be killed to 50% by Bisindolylmaleimide IX at 8 μM, we took the IC_50_ to be greater than 8 μM. Bisindolylmaleimide IX showed a modest cytotoxicity in these two cell types compared to other genotoxic drugs such as Itu (Figure 2C and 2D) [31]. Moreover, comparison of p53+/+ and p53−/− HCT116 cells revealed that p53 deficiency rendered modest resistance to the cytotoxicity of Bisindolylmaleimide IX (Figure 2D), suggesting that Bisindolylmaleimide IX has p53-dependent and -independent cytotoxic activities.

To test whether Bisindolylmaleimide IX has any effects on cell cycle checkpoints, we treated MEFs and HCT116 cells with Bisindolylmaleimide IX and found that the drug led to an increase in G2/M phase cells and a decrease in S phase cells (Figure 2E and 2F), suggesting that Bisindolylmaleimide IX activated G2/M and G1/S checkpoints. p53−/− HCT116 cells showed modestly decreased G2/M and G1 cell cycle arrest (Figure 2F), indicating that Bisindolylmaleimide IX causes cell cycle arrest in p53-dependent and -independent manners, with the latter likely attributable to the Atm-Chk2 pathway, which can be activated by Bisindolylmaleimide IX (Figure 2B).

### Bisindolylmaleimide IX shows strong cytotoxic effects on BCR-ABL positive K562 cells

**Figure 3:**
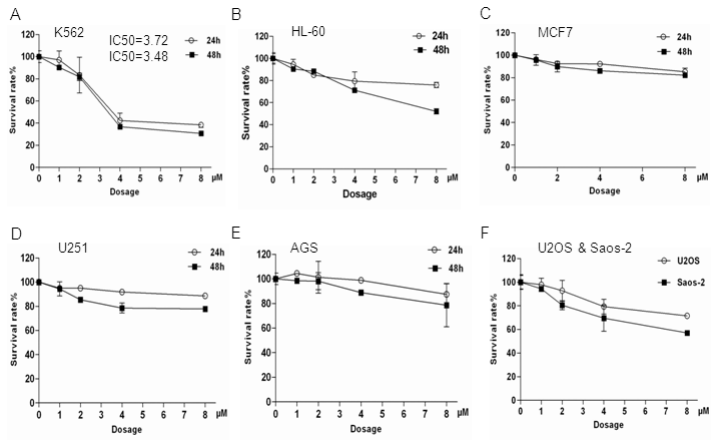
K562 cells were more sensitive to Bisindolylmaleimide IX-induced cell death compared to other cell lines tested These cell lines were treated with different doses of Bisindolylmaleimide IX for 24 or 48 hrs and the cell survival rates were determined by Wst-1 assays. **A.** Myeloid cell line K562. **B.** Myeloid cell line HL-60. **C.** Breast cancer cell line MCF7. **D.** Glioma cell line U251. **E.** Gastric cancer cell line AGS. **F.** Gastric cancer cell line MGC-803. **G.** Osteosarcoma cell lines U2OS and Saos-2.

We then tested the cytotoxicity of Bisindolylmaleimide IX in a number of cancer cell lines with different origins, hoping to identify the type of cancer that could be effectively targeted by Bisindolylmaleimide IX. It was found that K562, a BCR-ABL positive CML line, was highly sensitive to Bisindolylmaleimide IX (Figure 3A), compared to HL-60 (BCR-ABL negative leukemic line), breast cancer cell line MCF7, glioma cell line U251, gastric cancer cell lines AGS and MGC-803, osteosarcoma cell lines U2OS and Saos-2 (Figure 3B-3G). We also tested staurosporine and imatinib mesylate in these cells and found staurosporine showed strong cytotoxic effects in almost all these cell lines, whereas imatinib, like Bisindolylmaleimide IX, showed specificity to BCR-ABL positive cells (Supplementary Figure S3). Bisindolylmaleimide IX-induced cell death in K562 cells occurred by both necrosis and apoptosis, with necrosis the main form of cell death (Supplementary Figure S4). Since most of these cancer lines including K562 carry p53 mutations, these results suggest that Bisindolylmaleimide IX has p53-independent cytotoxic activity.

### BCR-ABL sensitizes cells to Bisindolylmaleimide IX-induced cell death and cell cycle arrest

**Figure 4:**
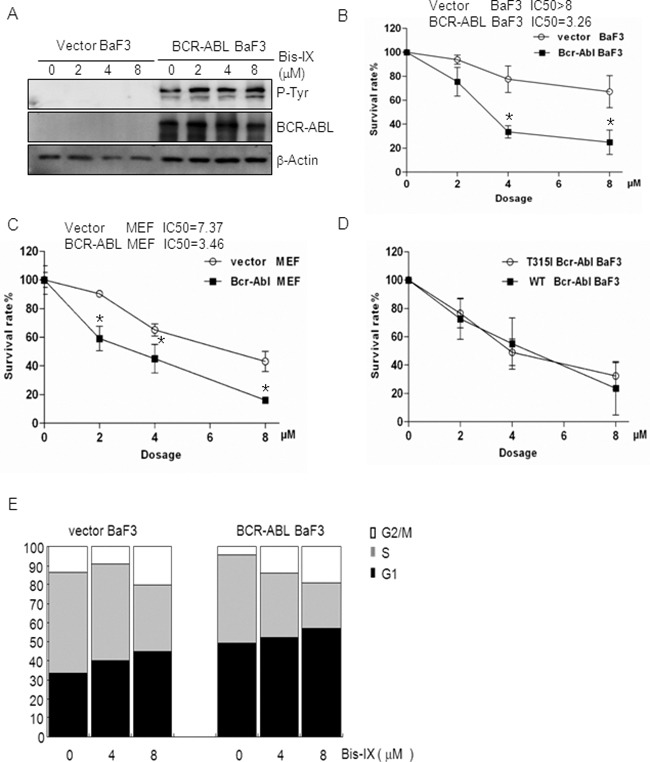
BCR-ABL sensitized cells to Bisindolylmaleimide IX-induced cell death and cell cycle arrest **A.** Western blot results showed that BCR-ABL was expressed but its activity was not inhibited by Bisindolylmaleimide IX. BaF3 cells infected with empty retrovirus or retrovirus expressing BCR-ABL were treated with different doses of Bisindolylmaleimide IX for 4 hrs. Activation and expression of BCR-ABL were determined by western blot. Activation of BCR-ABL was determined by anti-phospho-Tyr antibodies. **B.** BCR-ABL expressing BaF3 cells showed increased cell death rates in response to Bisindolylmaleimide IX. BaF3 cells carrying the vector or BCR-ABL were treated with different doses of Bisindolylmaleimide IX for 24 hrs and cell survival rates were determined by Wst-1 assays. N=3. *p<0.05 when the values of BCR-ABL positive BaF3 cells were compared to those of control cells. **C.** BCR-ABL expressing MEF cells showed increased cell death rates in response to Bisindolylmaleimide IX. MEF cells infected with the vector or BCR-ABL-expressing retrovirus were treated with different doses of Bisindolylmaleimide IX for 24 hrs and cell survival rates were determined by Wst-1 assays. N=3. *p<0.05 when the values of BCR-ABL positive MEFs were compared to those of control MEFs. **D.** T315I mutant BCR-ABL also sensitized BaF3 cells to the cytotoxicity of Bisindolylmaleimide IX. BaF3 cells expressing BCR-ABL or T315I BCR-ABL were treated with different doses of Bisindolylmaleimide IX for 24 hrs and cell survival rates were determined by Wst-1 assays. **E.** BCR-ABL expressing BaF3 cells showed increased cell cycle arrest compared to vector-infected BaF3 cells in response to Bisindolylmaleimide IX. BaF3 cells carrying the vector or BCR-ABL were treated with different doses of Bisindolylmaleimide IX for 24 hrs and cell cycle profiles were determined by FACS analysis. The values are average of three repeated experiments. Right panels: quantitative data for S and G2/M phases.

The above findings suggest that Bisindolylmaleimide IX might have the potential to treat CML, a disease with 95% of the cases caused by BCR-ABL [3]. To validate these findings, we made use of BaF3 cells [37], a p53 defective cell line that has been widely used to study BCR-ABL function and to test the effects of anti-CML drugs [38]. We expressed BCR-ABL in BaF3 cells using a retrovirus vector and introduced an empty retroviral vector as a control (Figure 4A). We treated these cells with different doses of Bisindolylmaleimide IX for 24 hrs and cell survival rates were determined by Wst-1 assay. The results clearly showed that BCR-ABL expression sensitized the cells to the cytotoxic effect of Bisindolylmaleimide IX (Figure 4B). Ectopic expression of BCR-ABL in MEF cells also sensitized the cells to Bisindolylmaleimide IX-induced cell death (Figure 4C). We also tested the Imatinib-resistant T315I mutant BCR-ABL and found that this mutant rendered BaF3 cells similar sensitivity to Bisindolylmaleimide IX as BCR-ABL (Figure 4D). These results support the concept that Bisindolylmaleimide IX may be useful to treat BCR-ABL positive leukemia, including CML that is resistant to Imatinib, nilotinib, and dasatinib.

We also compared the cytotoxic effects of Bisindolylmaleimide IX to two other DNA topoisomerase inhibitors, doxorubicin and teniposide, and found that BCR-ABL positive BaF3 cells showed similar survival rate as control cells in response to doxorubicin, but increased survival in response to teniposide (Supplementary Figure S5), suggesting that Bisindolylmaleimide IX's selective cytotoxicity to BCR-ABL positive cells is not shared by other DNA topoisomerase inhibitors. We then compared the cytotoxic effects of various bisindolylmaleimide derivatives on BCR-ABL expressing cells, hoping to identify the most potent one(s). We treated BaF3 cells expressing BCR-ABL with different doses of each of the eleven available compounds (bisindolylmaleimide I to XI (Supplementary Figure S1)) for 24 hrs and determined the cell survival rates. It was found that Bisindolylmaleimide IX was the most potent one among these derivatives (Supplementary Figure S6). In addition, Enzastaurin (Supplementary Figure S1), a distant derivative, only showed a modest cytotoxic effect on BCR-ABL positive BaF3 and K562 cells (Supplementary Figure S7).

We then analyzed Bisindolylmaleimide IX-induced cell cycle arrest in BaF3 cells carrying an empty vector or expressing BCR-ABL and found that BCR-ABL-expressing BaF3 needed lower concentrations of Bisindolylmaleimide IX to activate G1/S checkpoint (compare the fold decrease/increase of different phases between the treated and untreated cells) (Figure 4E). Similarly, K562 cells were more sensitive to Bisindolylmaleimide IX-induced cell cycle arrest than HL-60 cells (Supplementary Figure S8). These results suggest that BCR-ABL sensitizes cells to Bisindolylmaleimide IX-induced cell cycle arrest.

### BCR-ABL enhances Bisindolylmaleimide IX-induced DNA damage and down-regulates DNA topoisomerase II

**Figure 5:**
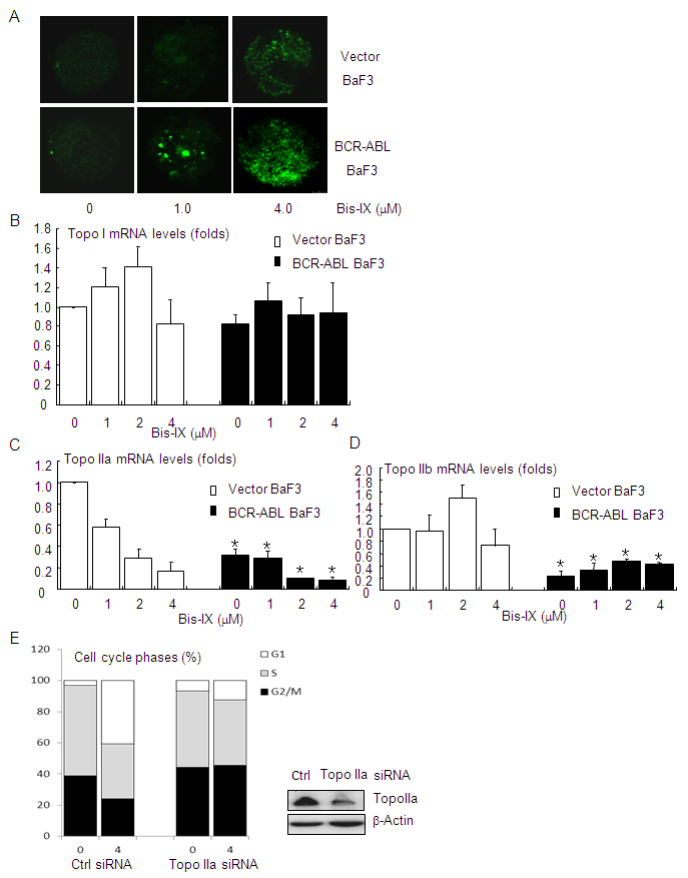
Bisindolylmaleimide IX induced increased DNA damage in BCR-ABL positive cells by suppressing the expression of topoisomerase II **A.** Bisindolylmaleimide IX induced an increase in DNA damage foci for γH2AX in BCR-ABL-expressing BaF3 cells. BaF3 cells infected with the vector or BCR-ABL-expressing retrovirus were treated with 1.0 or 4.0 μM Bisindolylmaleimide IX for 8 hrs and the foci formation was determined by immunofluorescent staining. **B.** BCR-ABL positive BaF3 cells showed similar levels of topoisomerase I mRNA as control cells. BaF3 cells carrying the vector or expressing BCR-ABL were treated with different doses of Bisindolylmaleimide IX for 8 hrs. The levels of topoisomerase I mRNA were determined by quantitative PCR. N=3. **C.** BCR-ABL positive BaF3 cells showed decreased levels of topoisomerase IIa mRNA, which were further suppressed by Bisindolylmaleimide IX treatment. BaF3 cells carrying the vector or expressing BCR-ABL were treated with different doses of Bisindolylmaleimide IX for 8 hrs. The levels of topoisomerase IIa mRNA were determined by quantitative PCR. N=3. *p<0.05 when the values of BCR-ABL positive BaF3 cells were compared to those of control cells at each dose. **D.** BCR-ABL positive BaF3 cells showed decreased levels of topoisomerase IIb mRNA. BaF3 cells carrying the vector or expressing BCR-ABL were treated with different doses of Bisindolylmaleimide IX for 8 hrs. The levels of topoisomerase IIb mRNA were determined by quantitative PCR. N=3. *p<0.05 when the values of BCR-ABL positive BaF3 cells were compared to those of control cells at each dose. **E.** BCR-ABL positive BaF3 cells with Topo IIa knockdown were refractory to Bisindolylmaleimide IX-induced cell cycle arrest at G2/M and S phases. Top panel: western blot results showed that Topo IIa was knocked down in BCR-ABL positive BaF3 cells. Bottom panel: the cell cycle profiles of BCR-ABL positive BaF3 cells with Topo IIa knockdown in response to Bisindolylmaleimide IX.

CML cells are known to be genetically unstable due to BCR-ABL. This is the reason behind increased mutagenesis of *BCR-ABL* in these cells [9, 10]. We found that Bisindolylmaleimide IX induced increased numbers of γH2AX foci in BaF3 cells expressing BCR-ABL compared to control BaF3 cells (Figure 5A), suggesting that BCR-ABL promoted Bisindolylmaleimide IX-induced DNA damage. We then analyzed the expression of DNA topoisomerases, the targets of Bisindolylmaleimide IX, in BaF3 cells carrying the vector or BCR-ABL. Quantitative PCR analysis revealed that Topo I was expressed at similar levels in BaF3 cells carrying BCR-ABL or the vector, which was not significantly altered by Bisindolylmaleimide IX treatment (Figure 5B). On the other hand, BCR-ABL positive BaF3 cells expressed decreased levels of Topo IIa, which were further repressed by Bisindolylmaleimide IX treatment (Figure 5C), and decreased levels of Topo IIb, which was not affected by Bisindolylmaleimide IX treatment (Figure 5D). These results indicate that BCR-ABL suppresses the expression of Topo IIa and IIb and that Bisindolylmaleimide IX may directly target Topo IIa. Decreased levels of topoisomerases are likely to sensitize the cells to Bisindolylmaleimide IX by increasing the drug-target ratio in these cells. These results, together with our finding that Bisindolylmaleimide IX is an inhibitor of DNA topoisomerase (Figure 1D), suggest that Topo IIa may be a target of Bisindolylmaleimide IX. Indeed, we found that knockdown of Topo IIa with siRNA rendered BCR-ABL positive cells resistance to Bisindolylmaleimide IX-induced cell cycle arrest (Figure 5E).

One important cause of genome instability in CML cells is accumulation of ROS [9, 39–41], which are produced via mechanisms including superoxide dismutase and NADPH oxidase [9, 42]. We treated BCR-ABL expressing BaF3 cells with Bisindolylmaleimide IX and found that ROS levels were not significantly altered (Supplementary Figure S9A). On the other hand, BaF3 cells carrying the empty vector showed lower levels of ROS (Supplementary Figure S9A), confirming that BCR-ABL promoted ROS production. However, depletion of ROS with N-Acetyl Cysteine (NAC), a ROS scavenger, showed an insignificant rescuing effect on Bisindolylmaleimide IX-induced cell cycle arrest or cell death rate in BCR-ABL positive BaF3 cells (Supplementary Figure S9B and data not shown), suggesting that ROS do not play an role in Bisindolylmaleimide IX-induced DNA damage response.

### BCR-ABL sensitizes cells to Bisindolylmaleimide IX-induced cell death via the oncogene addiction pathway

**Figure 6:**
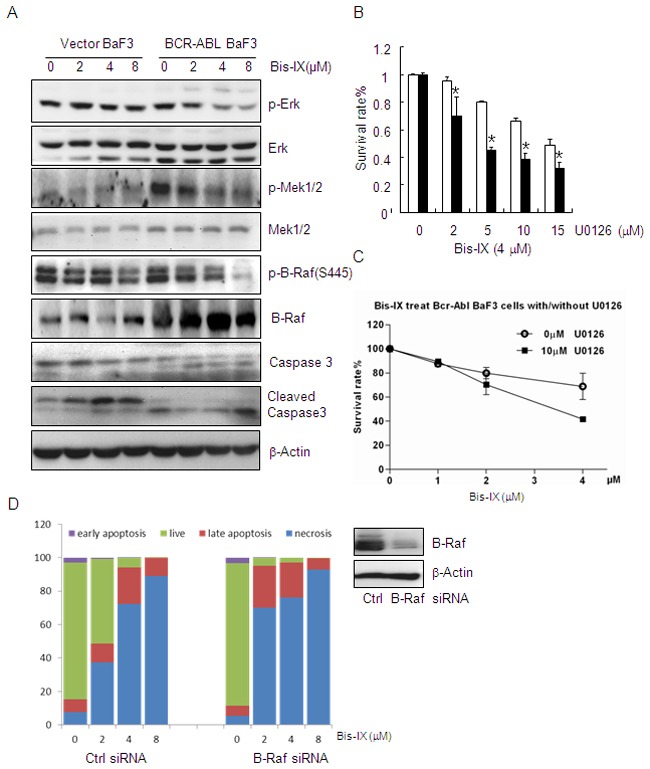
Bisindolylmaleimide IX shows increased cytotoxicity to BCR-ABL positive cells by inhibiting Raf-Erk signaling **A.** Bisindolylmaleimide IX inhibited Raf-Erk activation in BCR-ABL positive BaF3 cells. BaF3 cells infected with empty retrovirus or retrovirus expressing BCR-ABL were treated with different doses of Bisindolylmaleimide IX for 4 hrs. Activation and expression of Erk, Mek1, and Raf were determined by western blot. **B.** Inhibition of Erk decreased the cell survival rates in BaF3 cells expressing BCR-ABL compared to control cells. N=3. *p<0.05 when the values of BCR-ABL positive BaF3 cells were compared to those of control cells at each dose. **C.** Inhibition of Erk also enhanced Bisindolylmaleimide IX-induced cell death in BCR-ABL expressing BaF3 cells. N=3. *p<0.05 when the values of BCR-ABL positive BaF3 cells were compared to those of control cells at each dose. **D.** Knockdown of B-Raf lowered the survival of BCR-ABL expressing BaF3 cells in response to Bisindolylmaleimide IX. N=3. Right panel: Western blot results showing that B-Raf was knocked down in these cells.

The above findings suggest that there exist p53-independent mechanisms by which BCR-ABL sensitizes the cells to Bisindolylmaleimide IX, as BaF3 and K562 cells express mutant p53. We found that Bisindolylmaleimide IX showed negligible inhibitory effect on BCR-ABL activity in vivo and it needed 45 μM to inhibit BCR-ABL in *in vitro* kinase assays (Figure 4A and Table 1), suggesting that Bisindolylmaleimide IX is not a strong inhibitor of BCR-ABL per se. Bisindolylmaleimide IX may target the downstream molecules of BCR-ABL, especially the one (s) that renders the cells addiction to BCR-ABL, e.g., Erks [5, 6] [43]. We found that Bisindolylmaleimide IX inhibited Erk activation in BCR-ABL-expressing BaF3 cells but not in control cells (Figure 6A). Functionally, we found that inhibition of Erk with U0126 induced increased cell death rates in BCR-ABL expressing BaF3 cells than control cells (Figure 6B), confirming that Erk1/2 play an important pro-survival role in these cells [44, 45]. Since inhibition of Erk activation by Bisindolylmaleimide IX is not complete, we tested combination of Bisindolylmaleimide IX and U0126 and found that this further increased cell death rates in BCR-ABL-expressing BaF3 cells (Figure 6C). These results suggest that Erk activity may play a role in mediating the cytotoxic effect of Bisindolylmaleimide IX in BCR-ABL-expressing BaF3 cells [46, 47].

**Table 1:** Inhibitory effects of Bisindolylmaleimide IX on a variety of kinases

enzyme compounds	IC50							
	Aurora A	B-Raf	IKKβ	Jak2	SYK	BCR-ABL	ERK1	MEK1
Bis-IX (μM)	>45	1.14±0.11	0.48±0.005	9.68±0.79	> 45	> 45	> 45	> 45
Stauprorine (nM)	13.71±2.71	3.37±0.17	1.54±0.12	0.76±0.04	1.16±0.08	N.D.	N.D.	N.D.
Imatinib (μg/ml)	N.D.	N.D.	N.D.	N.D.	N.D.	0.023±0.003	N.D.	N.D.

N.D. : not determined

We then attempted to identify the target of Bisindolylmaleimide IX in the BCR-ABL-Erk1/2 pathway, which include Raf, Mek, and Erk [43]. We found that Bisindolylmaleimide IX inhibited phosphorylation of B-Raf, as well as activation of Mek1, in a more sensitive manner in BCR-ABL positive BaF3 cells than control cells (Figure 6A). A previous study reported that Raf molecules could be activated by PKC, which could be inhibited by Bisindolylmaleimide IX [48]. In addition, *in vitro* kinase assays showed that Bisindolylmaleimide IX was a potent inhibitor of B-Raf, the main regulator of Mek1-Erk1/2 among the Raf homologs [49], with an IC_50_ of 1.14 μM (Table 1), without inhibiting Mek1 or Erk1 activity, with IC_50_ values greater than 45 μM (Table 1). We found that DNA topoisomerase inhibitor doxorubicin and teniposide did not affect the phosphorylation of B-Raf in BCR-ABL positive BaF3 cells (Supplementary Figure S10), which may explain why cytotoxicity of these two drugs is not affected by BCR-ABL (Supplementary Figure S5).

To test the role of B-Raf in Bisindolylmaleimide IX-induced cytotoxicity in BCR-ABL positive BaF3 cells. We knocked down B-Raf and found this greatly sensitized the cells to Bisindolylmaleimide IX-induced cell death (both apoptosis and necrosis) (Figure 6D), whereas ectopic expression of constitutive active B-Raf (E600) rendered resistance to the drug (Supplementary Figure S11). Note that Bisindolylmaleimide IX–induced cell death is mainly necrosis (Figure 6D). These results indicate that B-Raf play an important role in Bisindolylmaleimide IX-induced cell death in BaF3 cells. However, Bisindolylmaleimide IX–induced DNA damage response is not caused by its inhibition on B-Raf, as B-Raf knockdown did not induce foci formation for γH2AX or p-ATM or induction of p53 (Supplementary Figure S12). Moreover, B-Raf knockdown compromised Bisindolylmaleimide IX–induced DNA damage response, manifested by a decrease in foci formation for γH2AX and p-ATM and p53 induction (Supplementary Figure S12).

### Bisindolylmaleimide IX is effective in treating CML-like disorders caused by BCR-ABL or T315I BCR-ABL

**Figure 7:**
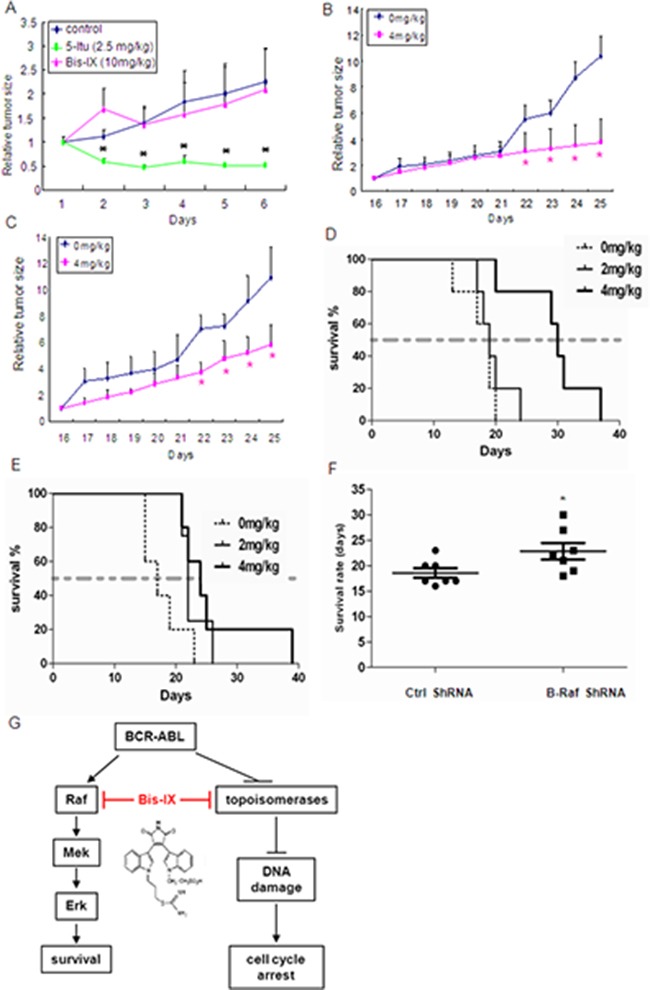
Bisindolylmaleimide IX was effective in treatment of leukemia-like disorders induced by BCR-ABL or T315I BCR-ABL **A.** Bisindolylmaleimide IX showed little effect on the size of tumors derived from HCT116 cells. The nude mice with tumor were treated with Bisindolylmaleimide IX, Itu, or solvent for different periods of time and the tumor size was measured every day. N=6. *p<0.05 when the tumor sizes with drug treatment were compared to those of untreated. **B.** Bisindolylmaleimide IX inhibited the growth of tumors derived from BCR-ABL-expressing BaF3 cells. The nude mice with tumor were treated with 4 mg/kg Bisindolylmaleimide IX or solvent for different periods of time and the tumor size was measured every day. The mean tumor volumes of treatment group were calculated at the final measurement, which were compared to that of vehicle-treated mice for statistical significance using Dunnett's test. N=8. *p<0.05 when the tumor sizes with drug treatment were compared to those of untreated. **C.** Bisindolylmaleimide IX inhibited the growth of tumors derived from T315I BCR-ABL-expressing BaF3 cells. The nude mice with tumor were treated with 4 mg/kg Bisindolylmaleimide IX or solvent for different periods of time and the tumor size was measured every day. The mean tumor volumes of treatment group were calculated at the final measurement, which were compared to that of vehicle-treated mice for statistical significance using Dunnett's test. N=8. *p<0.05 when the tumor sizes with drug treatment were compared to those of untreated at the same dose. **D.** Bisindolylmaleimide IX extended the lifespan of mice receiving BCR-ABL-expressing BaF3 cells. The nude mice were injected with BCR-ABL-expressing BaF3 cells. Three days later, these mice were treated with 2 or 4 mg/kg Bisindolylmaleimide IX or solvent every day and the lifespan of these mice were monitored. Each group contains 5 mice. **E.** Bisindolylmaleimide IX extended the lifespan of mice receiving T315I BCR-ABL-expressing BaF3 cells. The nude mice were injected with T315I BCR-ABL-expressing BaF3 cells. Three days later, these mice were treated with 2 or 4 mg/kg Bisindolylmaleimide IX or solvent every day and the lifespan of these mice were monitored. Each group contains 5 mice. **F.** Knockdown of B-Raf in BCR-ABL-expressing BaF3 cells with shRNA extended the lifespan of mice compared to control shRNA in response to Bisindolylmaleimide IX treatment. The nude mice were injected with BCR-ABL positive BaF3 cells that expressed control or B-Raf shRNA. Three days later, these mice were treated with 2 mg/kg Bisindolylmaleimide IX or solvent every day and the lifespan of these mice were monitored. Each group contains 7 mice. For B-Raf knockdown, see Supplementary results Figure S12. **G.** A diagram shows how Bisindolylmaleimide IX may treat CML. Bisindolylmaleimide IX inhibits DNA topoisomerases, which are down-regulated in BCR-ABL positive cells, to activate DDR and Atm-Chk2 to induce cell cycle arrest; and inhibits the Raf-Erk pathway, the BCR-ABL oncogene addiction pathway, to induce cell death.

We then tested the anti-cancer potential of Bisindolylmaleimide IX *in vivo*. We first treated nude mice carrying tumors derived from HCT116 with Bisindolylmaleimide IX at the dose of 10 mg/kg or ITU (2.5mg/kg) for 7 days. We found that the tumor size on Bisindolylmaleimide IX-treated mice did not go down and the body weights of the mice were not affected either, whereas Itu greatly inhibited tumor growth (Figure 7A and data not shown), suggesting that Bisindolylmaleimide IX is not effective in fighting HCT116-induced colorectal tumors. More importantly, these results suggest that Bisindolylmaleimide IX has little side effect even at high doses. We then tested whether Bisindolylmaleimide IX might be effective in treating BCR-ABL positive CML. We used BCR-ABL expressing BaF3 cells, which were able to form a tumor when implanted subcutaneously in nude mice, whereas BaF3 cells carrying a vector did not. In this solid tumor model, 4 mg/kg of Bisindolylmaleimide IX significantly inhibited growth of the tumors (Figure 7B). Moreover, 4 mg/kg of Bisindolylmaleimide IX also significantly inhibited growth of tumors derived from BaF3 cells expressing T315I BCR-ABL (Figure 7C).

Intravenous injection of BCR-ABL-expressing BaF3 cells, but not the cells carrying the empty vector, could kill nude mice 3 weeks after injection due to development of CML-like disorder. Bisindolylmaleimide IX was able to extend the lifespan of the nude mice carrying intravenously injected BCR-ABL positive BaF3 cells (Figure 7D), as well as the nude mice carrying intravenously injected T315I BCR-ABL positive BaF3 cells (Figure 7E). In the settings, knockdown B-Raf in BCR-ABL positive BaF3 cells significantly extended the lifespan of the nude mice compared to the control mice, in response to treatment with Bisindolylmaleimide IX (Figure 7F), confirming that B-Raf determines the sensitivity to Bisindolylmaleimide IX in vivo. These results show that Bisindolylmaleimide IX is effective in treating leukemia-like disorders induced by BCR-ABL or drug resistant BCR-ABL mutant in mouse models.

## DISCUSSION

Chemotherapy faces many obstacles including drug resistance and side effects. For example, many of the CML patients develop overt resistance to imatinib due to mutations in BCR-ABL, and T315I mutation appears to be resistant to Imatinib and the next generation drugs nilotinib and dasatinib [7, 8, 14]. Here by searching for novel genotoxic drugs, we identified Bisindolylmaleimide IX, which was shown to be a DNA topoisomerase inhibitor, as a drug candidate against BCR-ABL or T315I BCR-ABL positive cells and cancer (solid or leukemia), while it was less effective in other cell types tested. This specificity to BCR-ABL positive cells is attributable to two unrelated properties of Bisindolylmaleimide IX (Figure 7G). Firstly, as an inhibitor for topoisomerase especially Topo IIa, Bisindolylmaleimide IX induces increased DNA damage and increased cell cycle arrest, which are likely due to decreased expression of topoisomerase II isoforms in BCR-ABL positive cells. Secondly, as an inhibitor of B-Raf, Bisindolylmaleimide IX disrupts the oncogene addiction pathway of BCR-ABL and induces increased cell death (Figure 7G). The second property is not shared by DNA topoisomerase inhibitors doxorubicin and teniposide. Thus, this study identified Bisindolylmaleimide IX as a potential chemotherapeutic agent for CML, including drug-resistant CML.

Bisindolylmaleimide IX is one of many bisindolylmaleimide derivatives, which were initially developed as inhibitors for the PKC family [25]. Recently, efforts have been made in developing Bisindolylmaleimide derivatives into anti-cancer drugs [27, 50]. The best-studied is PKCβ inhibitor Enzastaurin, which has been tested alone or in combination with other chemotherapeutic drugs to treat glioma, lung cancer, ovary cancer, T and B cell lymphomas, colorectal cancer and other types of cancer [26, 50]. Enzastaurin is believed to execute its anti-cancer activity by inhibiting synthesis of VEGF and/or inhibiting the Akt1 signaling pathway [26, 50]. Our present study reveals that Bisindolylmaleimide IX is more effective than Enzastaurin and other bisindolylmaleimide derivatives in killing BCR-ABL positive cells.

We show for the first time that Bisindolylmaleimide IX is a DNA topoisomerase inhibitor that can cause cell cycle arrest and apoptosis. Previous studies have shown that Bisindolylmaleimide IX could induce apoptosis by cleaving anti-apoptotic protein Mcl-1 in chronic lymphocytic leukemia (CLL) cells, a disease not caused BCR-ABL, and induce apoptosis in HL-60 [51–53]. However, there are studies showing that Bisindolylmaleimide IX inhibits apoptosis in cells including thymocytes [54, 55]. We tested many cancer cell lines and found Bisindolylmaleimide IX in general showed a modest cytotoxic activity, with the exception of BCR-ABL positive cell lines. One possible explanation why genotoxic drug Bisindolylmaleimide IX fails to efficiently kill these cancer cells is that Bisindolylmaleimide IX, as a small molecule compound, may activate pro-survival genes or pathways [25, 56].

One reason why BCR-ABL-induced CML-like disorders are more susceptible to Bisindolylmaleimide IX is that this agent causes increased cells cycle arrest due to increased DNA damage. BCR-ABL down-regulates the expression of topoisomerase IIa and IIb, the levels of which are known to determine cell sensitivity to topoisomerase inhibitors such as Bisindolylmaleimide IX. Thus, this study identified another mechanism by which BCR-ABL affects genome stability [57]. Another reason is that Bisindolylmaleimide IX inhibits the Raf-Erk pathway, the oncogene addictive pathway in BCR-ABL positive cells [28, 45]. Bisindolylmaleimide IX targets the Raf-Erk pathway by directly inhibiting B-Raf, thus inducing greater cell death of BCR-ABL expressing cells, a mechanism that does not exist in other cancer lines tested.

In summary, this study suggests that Bisindolylmaleimide IX has the potential to treat BCR-ABL positive leukemia including CML that is refractory to imatinib, nilotinib, and dasatinib. Unlike BCR-ABL kinase inhibitor drugs like imatinib, Bisindolylmaleimide IX targets topoisomerase and B-Raf and takes advantage of pro-DNA damage activity of BCR-ABL and the oncogene addiction Raf-Erk pathway in BCR-ABL positive CML cells. Moreover, the effective doses of Bisindolylmaleimide IX show little side effect in vivo. Lastly, Bisindolylmaleimide IX may be worth testing against other cancer types that have common features as CML.

## MATERIALS AND METHODS

### Ethics statement

Animal experiments in this study, including BALB/cASlac nude mice and normal C57B/6 mice were carried out in accordance with recommendations from the National Research Council Guide for Care and Use of Laboratory Animals, with the protocols approved by the Institutional Animal Care and Use Committee of Shanghai, China [SYXK (SH) 2011–0112].

### Cell culture, retrovirus infection, and siRNA knockdown

The primary mouse embryo fibroblast (MEF) cells (from C57B/6 mice) were generated in the laboratory as described previously [29]. The human colon cancer cell lines HCT116 (p53+/+) and HCT116 (p53−/−) were a gift from B. Vogelstein's lab. These cells as well as human breast cancer cell line MCF7, human gastric cancer cell line AGS, human gastric cancer cell line MGC-803, human astrocytoma cell line U251, osteosarcoma cell line U2OS and HEK293T were cultured in DMEM. The human osteosarcoma cell line Saos-2 was cultured in McCoy's 5A. All these cells were cultured in the presence of 10% fetal bovine serum (Hyclone, Logan, UT, USA), 1% penicillin/streptomycin at a humidified atmosphere with 5% CO_2_.

BaF3 cells (murine Pro-B cells) were purchased from the Peking Union Cell Bank of Chinese Academy of Medical Sciences. BaF3 cells were infected with retroviruses expressing BCR-ABL, T315I BCR-ABL or empty vector. K562, HL60 cells and stable BaF3 cells expressing BCR-ABL or T315I BCR-ABL were maintained in RPMI 1640 medium supplemented with 10% fetal bovine serum, 2 mM glutamine, and 50 u/ml penicillin/streptomycin in a humidified incubator with 5% CO2. All BaF3 cells were cultured in the presence of 2 ng/ml murine IL-3. Ectopic expression of B-Raf (E600) was mediated by pMSCV-based retroviruses. pMSCVpuro retroviruses were used as a negative control.

To knock down TopIIa or B-Raf, BaF3 cells (3 x 10^6^ cells per sample) were pelleted and resuspended in 100 μl room temperature Nucleofector Solution (LONZA, Swiss), which were then mixed with 100 nM siRNA (Origene, USA). The cell and siRNA mixture was transferred into certified cuvettes and electroporation was performed using Amaxa Nucleofector (LONZA, Swiss). The cells were then mixed with 500 μl of pre-equilibrated culture medium and then transferred into the 12-well plate to culture for 48 hrs. For shRNA-mediated B-Raf knockdown, shRNA was expressed by a lentivirus vector. Three short hairpin RNA (shRNA) interference sequences targeting B-Raf gene and a negative control sequence were synthesized and cloned into the pAVL4.3-shRNA-GFP vector. The negative control shRNA sequence is TTCTCCGAACGTGTCACGT, while the B-Raf shRNA sequences are GGAACTGTCTACAAGGGAA, GCCACAACTGGCAATTGTT, and CTCCCAATGTTC ATATAA. Lentiviruses were produced using a plasmid-based lentiviral packaging system (vector plasmid-psPAX2-pMD2.G (Addgene, Cambridge, MA, USA)) to transfect HEK293T cells.

### Quantitative PCR

Total RNA was isolated with Trizol reagent (Invitrogen) from BaF3 cells carrying the vector or expressing BCR-ABL, which were treated with Bisindolylmaleimide IX or solvent. The RNA was used to carry out reverse transcription using Roche Transcriptor First Strand cDNA Synthesis Kit. Quantitative PCR was carried out using the following primers. Topo I: forward, GAGGGAACCACCCCAAGATG, reverse, TCCAGG AGACCAGCCAAGTA; Topo IIa: forward, GGAGT CCGATGACGATGACG, reverse, TGCATCACGTC AGAGGTTGAG; Topo IIb: forward, ATGTAGGGAT GAACTGCAGGG, reverse, TTCTTGTCCCTCTGCTT GTTGT.

### Western blot analysis

Cells were lysed in TNEN buffer (50 mM Tris, 150 mM NaCl, 5 mM EDTA, 0.5% NP-40, and 0.1% Triton X-100) supplemented with 1 mM NaF, Na_2_VO_3_, 1 mM PMSF, and 1 μg/ml of aprotonin, leupeptin, and pepstatin A. Protein concentrations were determined using a Bio-Rad assay. Proteins were resolved by SDS-PAGE and transferred to polyvinylidene difluoride membranes (Millipore). Antibodies against p-Atm were from Abcam Corporation. Antibodies against p-Chk2 (Thr68), Chk2, p-p53 (Ser15), p53, Cleaved Caspase-3, phospho-p44/42 MAPK (Erk1/2) (Thr202/Tyr204), p44/42 MAPK (Erk1/2), Phospho-MEK1/2 (Ser221), MEK1/2 and p-B-Raf (Ser445) were purchased from Cell Signaling. Antibodies against Caspase 3, B-Raf and Atm were from Genetex. Antibodies against c-Abl, GAPDH, and β-actin were from Santa Cruz Biotechnology. Antibodies against p-Tyr were from Merck Millipore Corporation.

### Immunofluorescence histochemistry

MEFs or HCT116 cells were cultured on cover slips while Baf3 cells were cultured on slides coated by 0.1mg/ml poly-L-lysine, washed with phosphate-buffered saline (PBS) twice, and then fixed in 4% paraformaldehyde (PFA), which were permeabilized with 0.1% Triton X in PBS for 30 minutes at room temperature. The primary antibodies were diluted in PBS with 1% BSA (1:200). The slides were blocked (1% BSA in PBS) for 60 minutes at room temperature, incubated with primary antibodies overnight at 4°C, and followed by secondary antibody incubation for 60 min at RT. The slides were then mounted and observed under confocal microscope (Leica, Germany). Antibodies against γH2AX were from Bethyl Corporation.

### Cell cycle analysis

Cells were seeded on 35-mm dishes and cultured for 24 hrs, reaching 60–70% confluency. The cells were treated with different concentrations of Bisindolylmaleimide IX for 24 or 48 hrs, harvested by trypsinization, resuspended in 200 μl PBS, and fixed in 100% ethanol overnight at 4°C. The fixed cells were pelleted by centrifugation, resuspended in 800 μl PBS containing ribonuclease A (100 μg/ml) and incubated for 30 min at 37°C. Then 10 μl propidium iodide (PI, 4 mg/ml PBS) were added to the samples, which were assessed on a FACSCalibur flow cytometer using Cell Quest software (BD Bioscience).

### Cell death assay

To measure cell survival rates after Bisindolylmaleimide IX treatment, cells were plated at 1×10^4^ in 96 well plates and cultured for overnight, which were then treated with Bisindolylmaleimide IX for 48 hrs. Cell proliferation reagent WST-1 (Roche) was added to each well and was further incubated for 4 hrs at 37°C. The absorbance was measured against a control using microplate reader at 440 nm. The reference wavelength was 630 nm. To analyze the nature of cell death, an Annexin V-PI Apoptosis Detection Kit (BD Pharmingen, USA) was used. Cells were seeded into six wells and cultured for 24 hours after treatment with Bisindolylmaleimide IX. The cells were collected, washed twice and then resuspended in 100 μL of binding buffer, after which 5 μl of FITC Annexin V and 10 μl PI were added. Cells were gently vortexed and incubated for 15 minutes in the dark. Finally, cells were added 400 μl binding buffer and analyzed by flow cytometry (BD Biosciences, USA) within 1 hour.

### Kinase assay

The recombinant Auror, B-Raf, IKKβ, SYK, BCR-Abl, MEK1 and Erk1 proteins were expressed in Escherichia coli strain BL21-Codon Plus (DE3), purified by Ni-NTA Agarose (QIAGEN). The kinase assays were carried out with the Z'-LYTETM Kinase Assay kit using Ser/Thr3 Peptide substrate (Invitrogen). The reactions were carried out according to the Z-LYTE protocol and the results were read on an EnVision plate reader. All reactions were carried out in triplicate. Data were expressed as mean ±SEM.

### Carcinoma xenograft mouse models

Male BALB/cASlac-nude mice (4-week-old) were purchased from Shanghai Slac Laboratory Animal C. LTD. The mice were kept in the SPF mouse facility for 1 week before being inoculated with HCT116 cells. HCT116 cells were harvested by trypsinization, counted and resuspended in PBS at the concentration of 5×10^7^/ml. We injected subcutaneously 3×10^6^ cells to BALB/cASlac nude mice's back. After 2 weeks, the mice were divided into 3 groups (6 mice per group) and were treated with 5-Iodotubercidin (Itu), Bisindolylmaleimide IX or solvent (PBS). The mice were weighed and the size of tumor measured. The length (L) and width (W) of the tumor were measured with a digital caliper and expressed as tumor volume (0.5L×W^2^, mm^3^).

For BaF3 cells-induced tumor models, 1 x 10^6^ BaF3 cells expressing BCR-ABL, T315I BCR-ABL or empty vector were inoculated subcutaneously in the left thigh back or intravenously injected via the tail vein. For solid tumor models, the mice were injected with 4 mg / kg of Bisindolylmaleimide IX or solvent. The length (L) and width (W) of the tumor were measured by a digital caliper and expressed as tumor volume (0.5 L×W^2^, mm^3^). The body weight and the lifespan of the mice with intravenous injection of BaF3 cells were monitored every day. For B-Raf knockdown, lentivirus-mediated expressing shRNA against B-Raf was used.

### DNA topoisomerase I assay

Bisindolylmaleimide IX and other derivatives were added to MEF cultures and 24 hrs later, the cells were washed with PBS and harvested in TNE buffer. The control and Bisindolylmaleimide IX-treated cell extracts were directly used to test the topoisomerase activity using a kit from Topogen (Port Orange, USA) following the manufacturer's protocol, or pBluescript DNA as a template. The in vitro incubation lasted 30 min at room temperature. A 2% agarose gel was run to analyze DNA unwinding (Topogen) or 0.8% gel for pBluescript DNA.

### Data analysis

Statistical comparisons were performed using unpaired Student's two-tailed t-test, with p-values <0.05 considered statistically significant.

## SUPPLEMENTARY FIGURES


