# Supplementary material for: Identification of Bisindolylmaleimide IX as a potential agent to treat drug-resistant BCR-ABL positive leukemia
Source: Oncotarget. 2016 Aug 24;7(43):69945–60. doi: 10.18632/oncotarget.11566 (PMC5342526; doi:10.18632/oncotarget.11566)
Supplement: Supplementary file 1 [file oncotarget-07-69945-s001.pdf]

Identification of Bisindolylmaleimide IX as a potential agent to treat drug-resistant BCR-ABL positive leukemia

SUPPLEMENTARY FIGURES

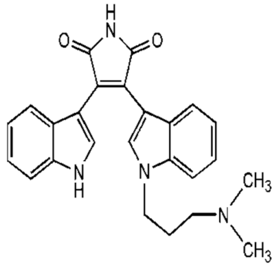

Bisindoylmaleimidine I

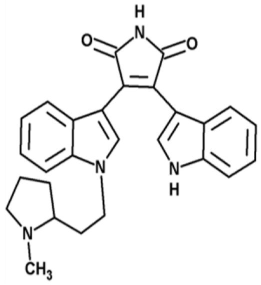

Bisindoylmaleimidine II

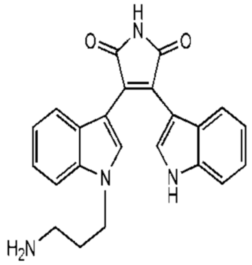

Bisindoylmaleimidine III

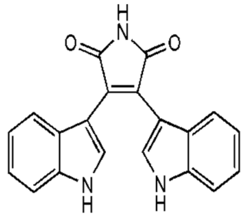

Bisindoylmaleimidine IV

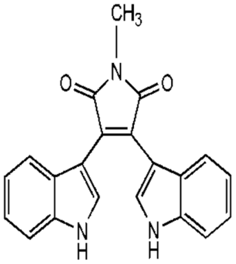

Bisindoylmaleimidine V

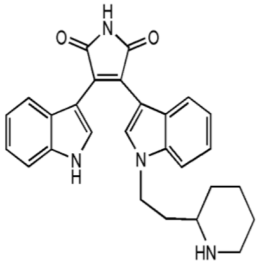

Bisindoylmaleimidine VI

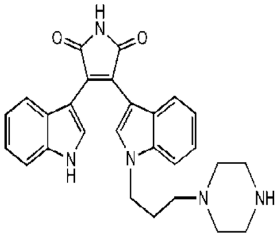

Bisindoylmaleimidine VII

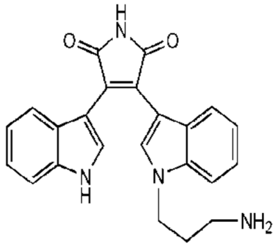

Bisindoylmaleimidine VIII

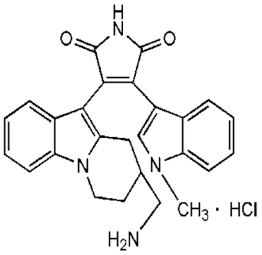

Bisindoylmaleimidine X

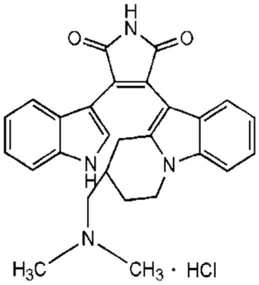

Bisindoylmaleimidine XI

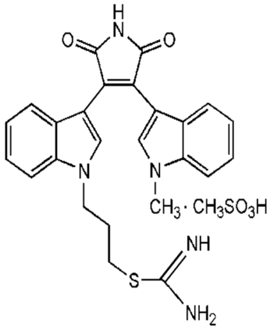

Bisindoylmaleimidine IX

Supplementary Figure S1: Chemical structures of the eleven Bisindolylmaleimide derivatives.

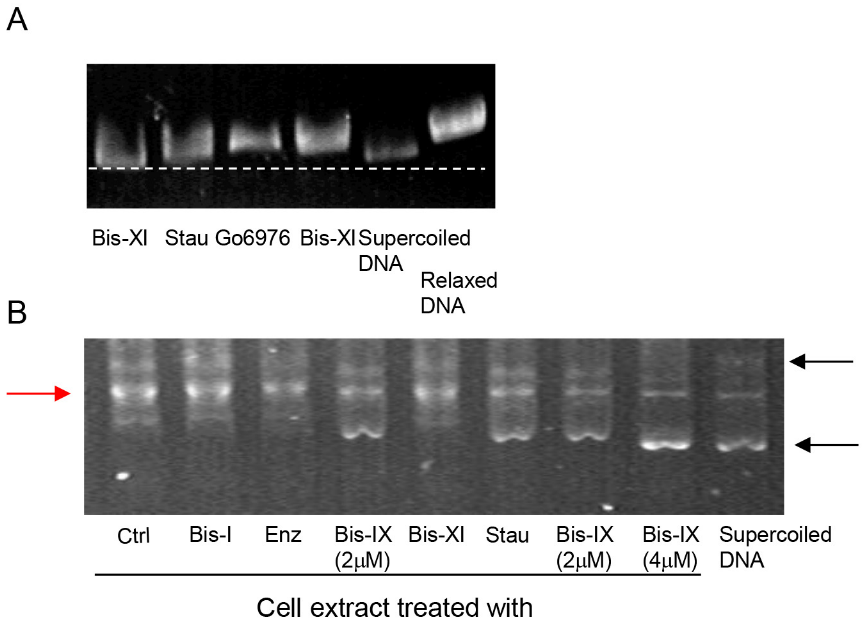

Supplementary Figure S2: The Topoisomerase inhibitor activity of Bisindolylmaleimide IX and other derivatives. **A.** Bisindolylmaleimide IX inhibited the Topoisomerase activity assayed with a Topoisomerase assay Kit. DNA samples from the Topoisomerase Assay Kit were incubated with cell lysates of MEFs, which were treated with Bisindolylmaleimide IX, I, Go6976, and Staurosporine (Stau). DNA samples were analyzed on agarose gels. **B.** Bisindolylmaleimide IX and several other derivatives inhibited the Topoisomerase activity assayed with pBluescript. Enzastaurin (Enz). Red arrow, relaxed DNA. Black arrows, supercoiled DNA.

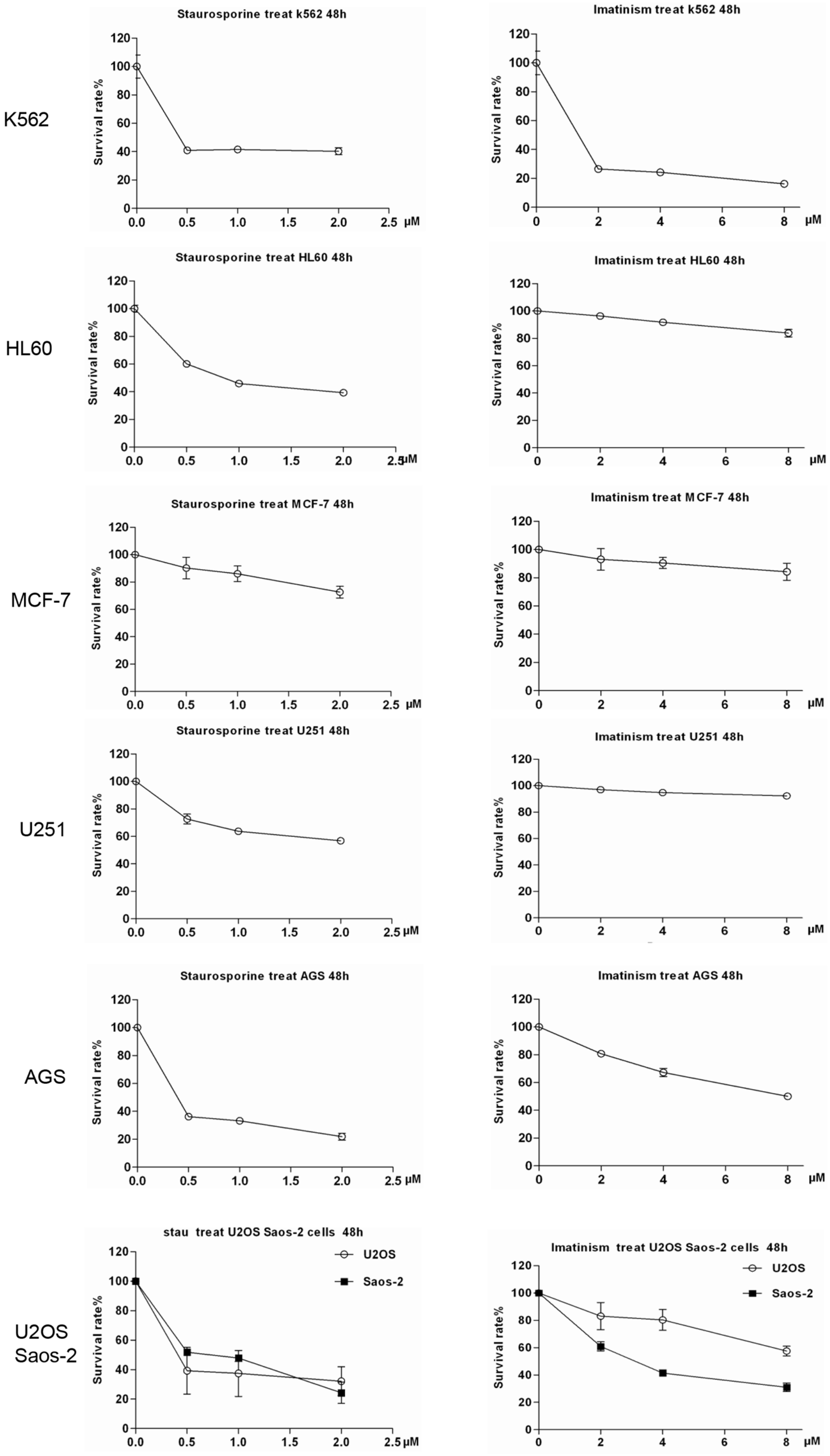

Supplementary Figure S3: The cytotoxic effects of staurosporine and Imatinib in various cell lines. These cells were treated with different doses of staurosporine and Imatinib for 24 hrs and cell survival rates were measured with Wst-1 assay.

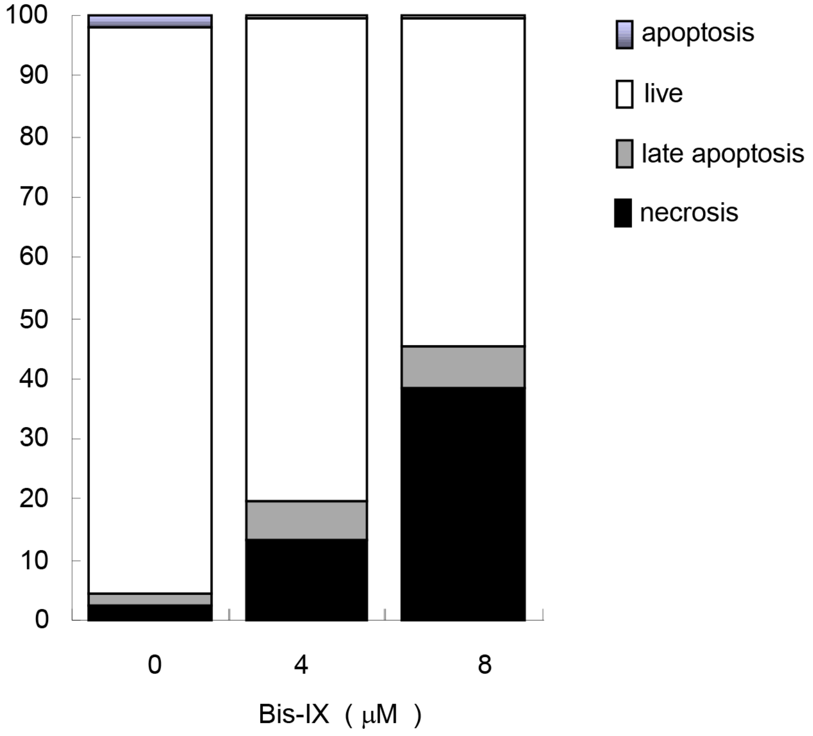

Supplementary Figure S4: Bisindolylmaleimide IX induces both apoptosis and necrosis in K562 cells. K562 cells were treated with different doses of Bisindolylmaleimide IX for 24 hrs. Cell necrosis and apoptosis rates were determined with Apoptosis and Necrosis Quantitation Kit Plus (Biotium Inc.).

A

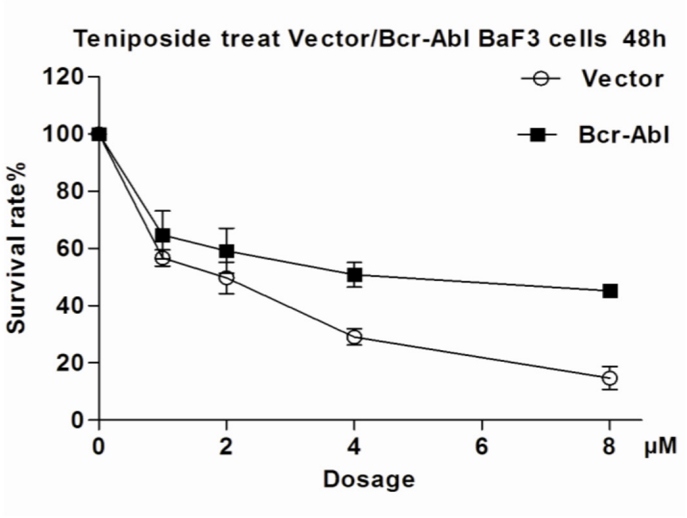

B

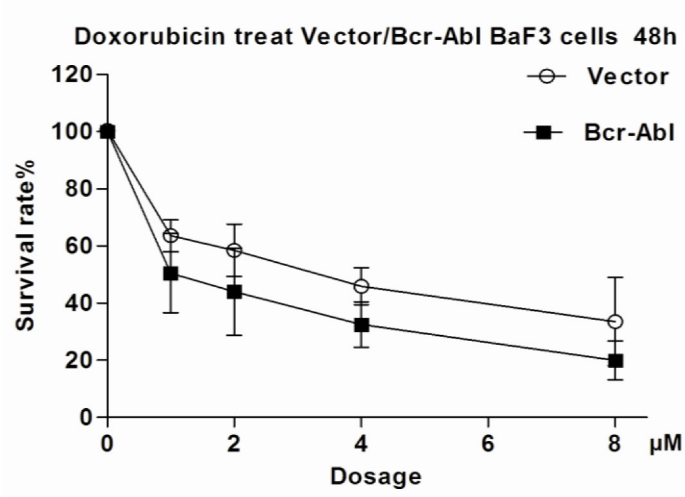

Supplementary Figure S5: The cytotoxic effects of two other DNA topoisomerase inhibitors, doxorubicin and teniposide in BCR-ABL positive and negative BaF3 cells. These two cell types were treated with different doses of doxorubicin and teniposide for 24 hrs and cell survival rates were measured with Wst-1 assay.

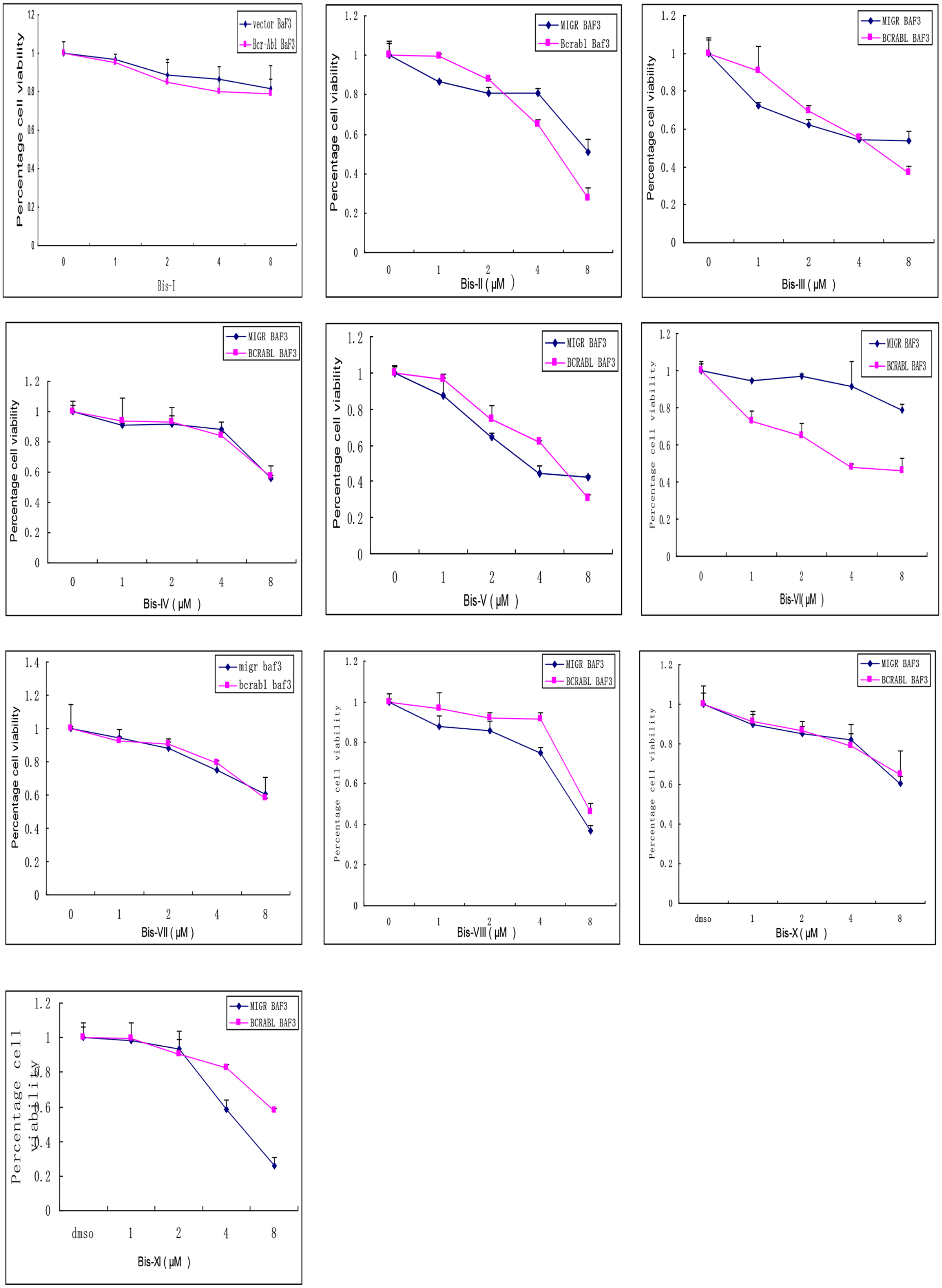

Supplementary Figure S6: The cytotoxic effects of various Bisindolylmaleimide derivatives (I to XI). BaF3 infected with the vector or BCR-ABL expressing retrovirus were treated with different doses of Bisindolylmaleimide derivatives for 24 hrs and the cell survival rates were determined by Wst-1 assay.

A

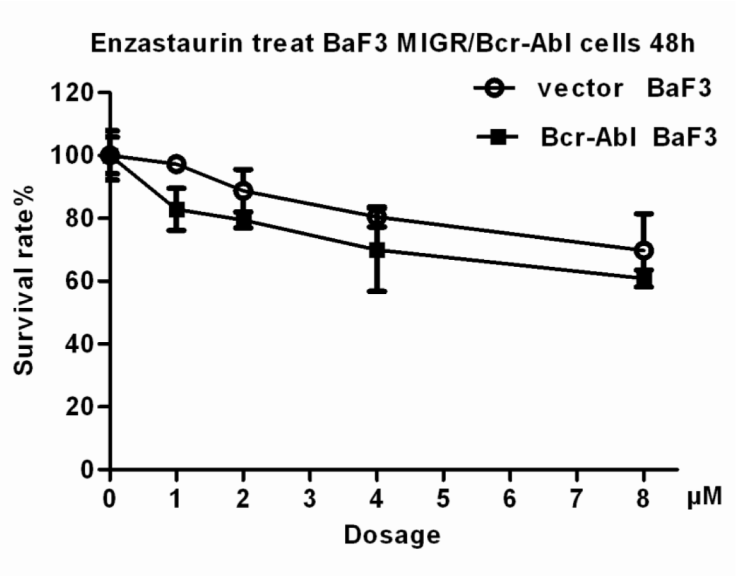

B

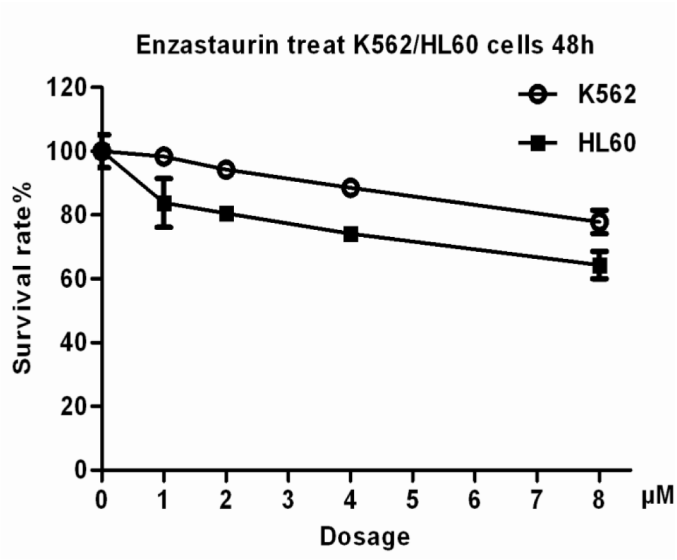

Supplementary Figure S7: The cytotoxic effects of Enzastaurin in K562 and BCR-ABL positive BaF3 cells. HL60, K562 **A.** and BaF3 cells **B.** infected with the vector or BCR-ABL expressing retrovirus were treated with different doses of Bisindolylmaleimide IX for 24 hrs and the cell survival rates were determined by Wst-1 assay.

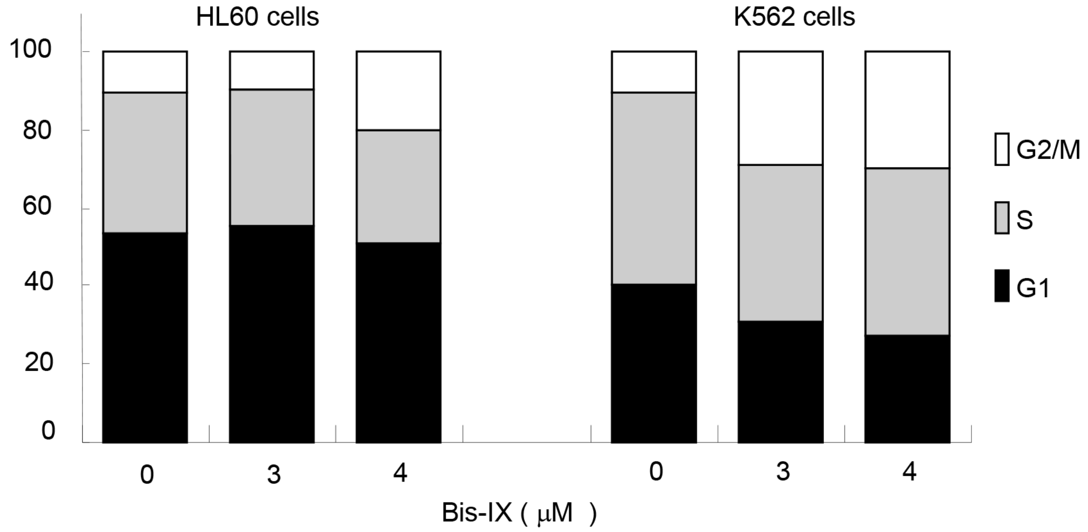

Supplementary Figure S8: K562 cells showed increased cell cycle arrest compared to HL-60 cells in response to Bisindolylmaleimide IX. HL-60 and K562 cells were treated with 2.5 μM Bisindolylmaleimide IX for 24 hrs and cell cycle profiles were determined by FACS analysis. The values are average of three repeated experiments.

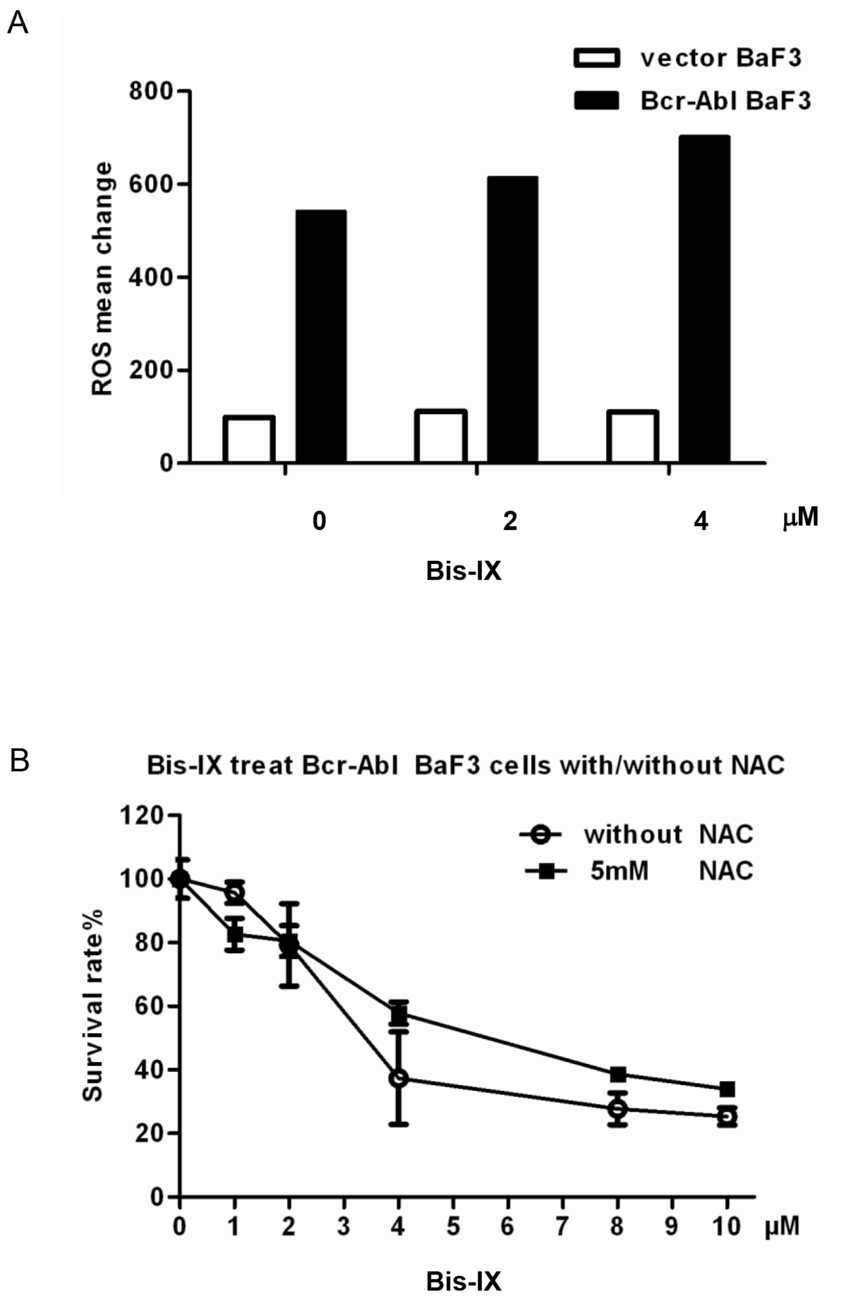

Supplementary Figure S9: BCR-ABL-induced ROS production is not involved in Bisindolylmaleimide IX-induced cell death or cell cycle arrest. A. Increased ROS levels in BCR-ABL positive cells. BaF3 cells infected with the vector or BCR-ABL-expressing retrovirus were treated with 2.0 or 4.0 μM Bisindolylmaleimide IX for 8 hrs and the cellular levels of ROS were determined by a ROS assay kit. **B.** Depletion of ROS with NAC did not significantly affect Bisindolylmaleimide IX-induced cell death. BaF3 cells infected with BCR-ABL-expressing retrovirus were pretreated with NAC for 2 hrs and then treated with different doses of Bisindolylmaleimide IX for 24 hrs and the cell survival rates were determined with Wst-1 assay.

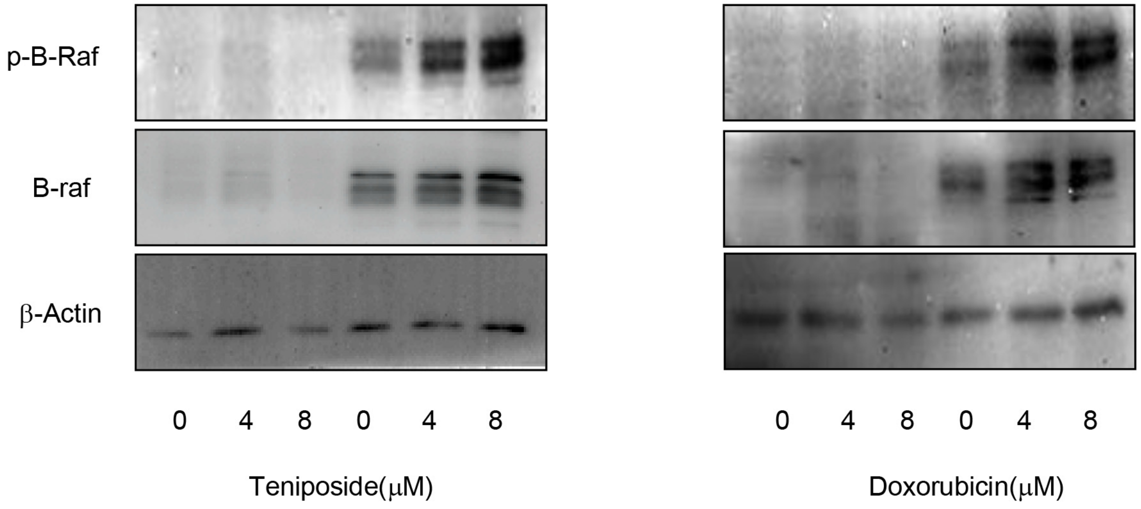

Supplementary Figure S10: BCR-ABL did not affect B-Raf activation in BaF3 cells in response to DNA topoisomerase inhibitor doxorubicin and teniposide. BCR-ABL positive BaF3 cells were treated with different doses of doxorubicin and teniposide and activation of B-Raf was determined by western blot analysis.

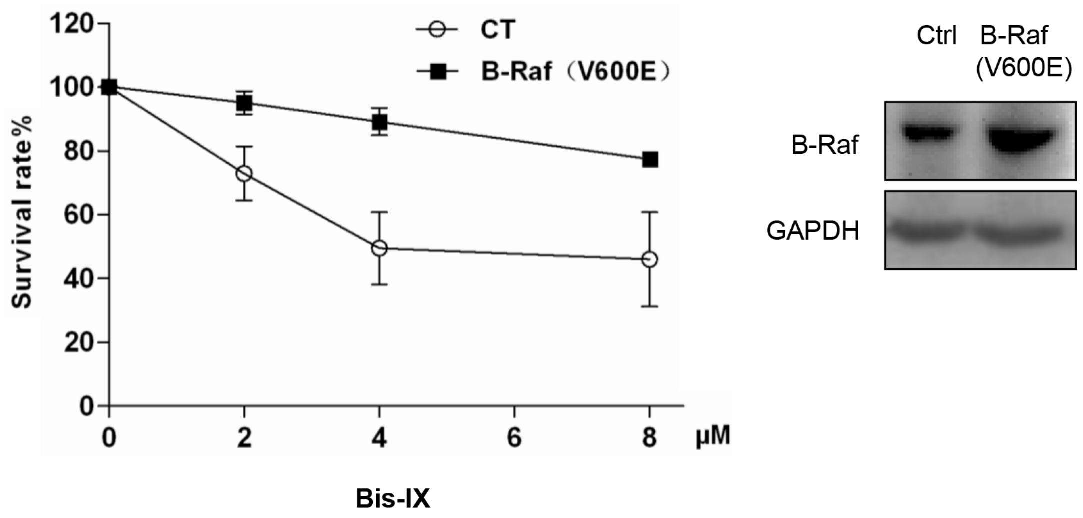

Supplementary Figure S11: Ectopic expression of constitutive active B-Raf increased the survival of BCR-ABL expressing BaF3 cells in response to Bisindolylmaleimide IX. N=3. Right panel: Western blot results showing that B-Raf was expressed in these cells.

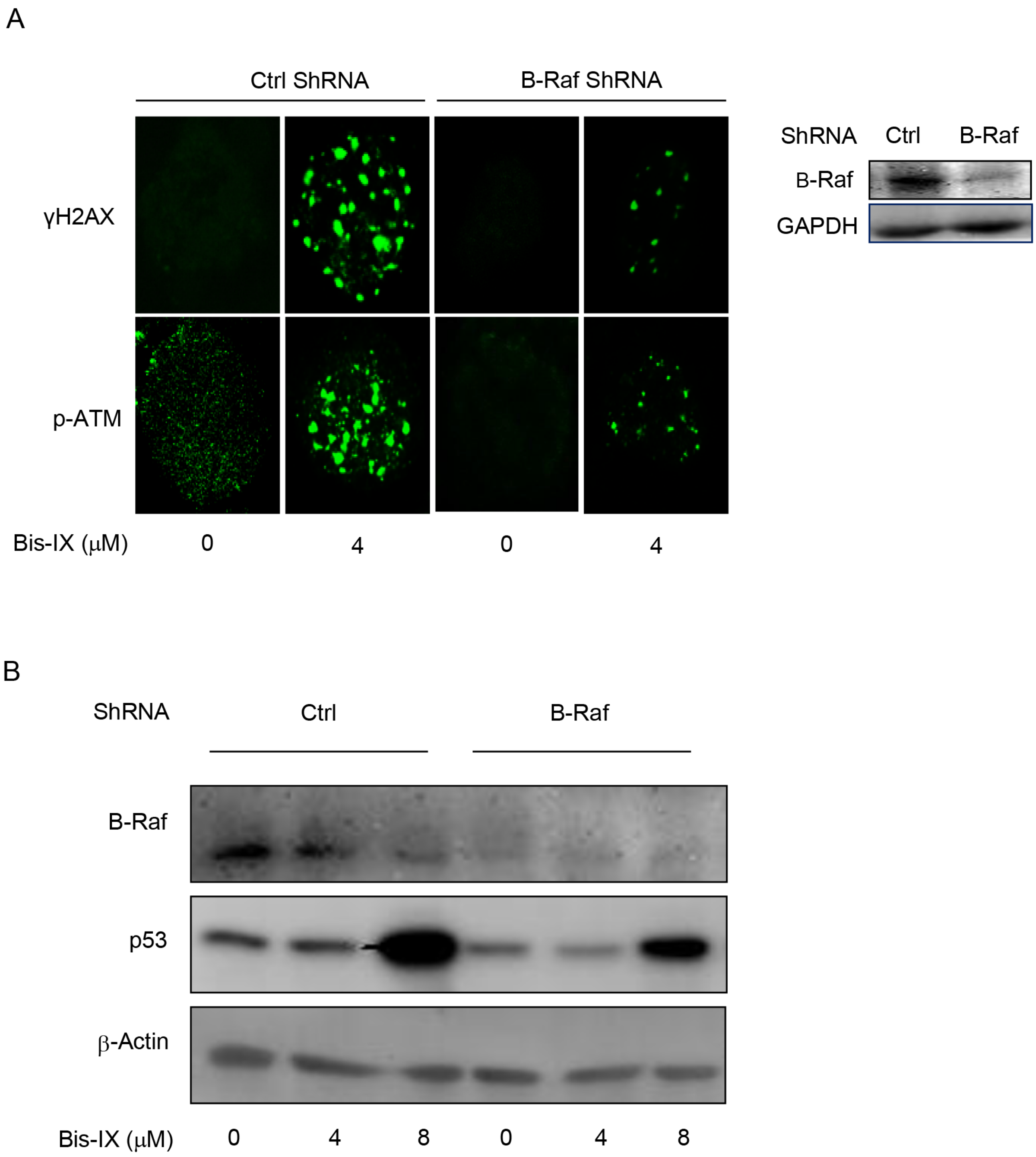

Supplementary Figure S12: Knockdown of B-Raf with shRNA did not affect baseline DNA damage response but compromised Bisindolylmaleimide IX-induced DNA damage response. The BCR-ABL positive Baf3 cells were infected with retroviruses expressing control or B-Raf shRNA, selected, and challenged with Bisindolylmaleimide IX. **A.** The effects of B-Raf knockdown on foci formation for γH2AX and p-ATM at the basal level and in response to Bisindolylmaleimide IX. Right panel: Western blot result showed that B-Raf was knocked down by shRNA. **B.** The effects of B-Raf knockdown on p53 induction at the basal level and in response to Bisindolylmaleimide IX. The cells were collected and western blot was used to determine the levels of p53.
